# Development and validation of machine learning models for predicting rehabilitation non-response after knee arthroplasty

**DOI:** 10.3389/fresc.2026.1930836

**Published:** 2026-09-18

**Authors:** Niclas J. Hubel, Ferdinand Prüfer, Walter Bily, David Schneider, Bernhard Holzner, Michael J. Fischer, Vincent Grote

**Affiliations:** 1 Austrian Center for Medical Innovation and Technology, Wiener Neustadt, Austria; 2 Health Outcomes Research Unit, University Hospital of Psychiatry II, Medical University of Innsbruck, Innsbruck, Austria; 3 Department of Orthopedics and Traumatology, Medical University of Graz, Graz, Austria; 4 Ludwig Boltzmann Institute for Rehabilitation Research, Vienna, Austria; 5 Vitrea Rehabilitation Center Kitzbühel, Kitzbühel, Austria; 6 Evaluation Software Development GmbH – ESD, Innsbruck, Austria

**Keywords:** arthroplasty, AutoML, health-related quality of life, minimal clinically importance difference, patient-reported outcomes, replacement knee

## Abstract

**Introduction:**

Structured rehabilitation after total knee arthroplasty (TKA) effectively improves health-related quality of life (HRQoL) and function, yet some patients fail to achieve minimal clinically important differences (MCIDs). Machine-learning (ML) based prediction models offer one way to identify those patients. We aim to develop and externally validate ML models to predict MCID-based non-response at discharge.

**Methods:**

Registry data from four Austrian rehabilitation centres (*N* = 5,463; 2018–2024), were used to predict non-response in a global (EQ-5D, *n* = 4,032), and an indication-specific patient-reported outcome measure (WOMAC, *n* = 4,213) and a performance test (Timed Up and Go Test - TUG, *n* = 3,354). Outcomes were dichotomized applying a distribution-based MCID and predicted using automated ML ensemble algorithms. Model performance was assessed in independent test data.

**Results:**

Models predicted non-response with good discrimination (AUCs: TUG 0.771, WOMAC 0.704, EQ-5D 0.770); and varying calibration (TUG: acceptable, WOMAC: good, EQ-5D: poor). Admission values were the most important predictors, with better values resulting in a higher likelihood of not achieving a MCID.

**Discussion:**

ML can predict MCID-based non-response after TKA rehabilitation using routinely collected admission data. Baseline status is a dominant driver, highlighting ceiling effects and the potential for bias, as well as the need for more precise outcome definitions and the search for suitable, independent predictors. Clinicians could use ML-based risk estimates to identify likely non-responders early, support personalized expectation management, and tailor therapy intensity or content. Translating these tools to practice will require prospective validation and integration into clinical workflows.

## 1 Introduction

Total knee arthroplasty (TKA) is the standard surgical option for symptomatic, end-stage knee osteoarthritis that remains refractory to non-surgical treatment and can effectively reduce pain, functional limitations and disability (1, 2). TKA is among the most frequently performed and cost-effective orthopaedic procedures, improving mobility and health-related quality of life and lowering long-term healthcare costs (3, 4). Contemporary care pathways combine fast-track perioperative management with structured rehabilitation, including early mobilization and multimodal biopsychosocial interventions, to prevent complications such as joint stiffness and muscle atrophy and to support rapid return to daily activities (5–8). Across inpatient, outpatient and home-based settings, early intensive rehabilitation yields substantial improvements in both patient-reported and performance-based outcomes with comparable long-term efficacy and safety (9, 10).

Recovery after TKA is multidimensional, and outcome assessment usually combines patient-reported outcome measures (PROMs), clinician-reported outcomes (CROMs) and performance-based measures. In clinical research and practice, “success” is commonly operationalized either as reaching an absolute post-treatment threshold, such as a patient acceptable symptom state, or as achieving a predefined amount of change in these scores. Meaningful change is often quantified using the minimal clinically important difference (MCID), defined as the smallest improvement in a PROM or CROM that patients perceive as beneficial and that would justify a change in clinical management (11). Correlations between PROMs and global satisfaction are typically weak to moderate (12, 13), although disease-specific, pain-focused PROMs tend to relate more strongly to satisfaction (14, 15).

Despite generally favourable outcomes in TKA rehabilitation, substantial heterogeneity in individual results persists. Between 10 and 30 percent of patients report dissatisfaction after TKA, and a similar-sized, but equally varying, subgroup fails to achieve an MCID or an acceptable symptom state (4, 10, 16–19). Registry-based work has shown that, depending on the instrument and baseline status, a non-trivial fraction of patients do not meet relative or absolute improvement thresholds after hip or knee replacement, and some cannot meet a predefined MCID at all because of scale limits (20–22). Identifying such non-responders early in the rehabilitation pathway could support targeted interventions, expectation management and more efficient allocation of rehabilitation resources (10, 23–25).

Prediction models based on routinely collected data offer one way to anticipate which patients are unlikely to achieve clinically meaningful gains. In arthroplasty, machine-learning algorithms applied to preoperative and perioperative data have been used to predict achievement of PROM-based MCIDs with fair-to-good discrimination and promising decision support potential (20, 21). In rehabilitation medicine, preliminary work suggests that combining PROMs and performance measures in machine-learning models can predict rehabilitation “success” with useful accuracy (26). Automated machine-learning (AutoML) frameworks further lower the barrier to model development by systematically exploring modelling choices and hyperparameters, but evidence in orthopaedic rehabilitation remains sparse, heterogeneous and focused predominantly on PROM-based outcomes (27–32). It is still unclear whether performance-based non-response can be predicted with similar accuracy, and how such models compare with traditional logistic regression.

In this study, we defined rehabilitation “success” individually for each outcome after a 3-week post-acute TKA rehabilitation program as achieving a MCID at discharge; patients not achieving the MCID were classified as non-responders. Using routinely collected data from four Austrian rehabilitation centres, we aimed to:

1. develop and externally validate machine-learning models to predict MCID-based non-response at discharge,
2. compare the predictive performance of models using PROM- versus performance-derived outcome definitions and to benchmark machine-learning models against baseline-only logistic regression, and
3. identify which baseline variables contribute most strongly to the prediction of non-response.

By clarifying for which outcome definitions clinically useful predictions can currently be achieved, and which factors drive model performance, we aim to inform future development of data-driven decision support for TKA rehabilitation.

## 2 Materials and methods

### 2.1 Design

We conducted a registry-based prediction-modelling study using routinely collected clinical data from orthopaedic rehabilitation centres in Austria. The primary aim was to develop and externally validate (i.e., test on independent, unseen data) machine-learning models for predicting non-response to post-acute rehabilitation after total knee arthroplasty (TKA).

The study methodology and reporting follow the Transparent Reporting of a Multivariable Prediction Model for Individual Prognosis or Diagnosis (TRIPOD) guideline and its AI (33) and cluster extension (34). Data management and conventional statistical analyses were performed in R version 4.4.1 (35). Machine-learning models were developed in Python using Jupyter Notebooks (see Supplementary Methods for details).

### 2.2 Ethical considerations

The study was conducted in accordance with the Declaration of Helsinki and applicable national data protection regulations. The protocol was approved by the Ethics Committee of the Medical University of Innsbruck (EK Nr: 1036/2025). All data were analyzed in anonymized form, and no individual patient can be identified from the reported results.

### 2.3 Setting and data source

The models were trained on data derived from the clinical registry of a large Austrian rehabilitation centre, which provides standardized post-acute inpatient rehabilitation for patients after knee arthroplasty under the Austrian social insurance system (36, 37) (see supplementary for more details). The registry systematically records sociodemographic, clinical, and rehabilitation-related variables at admission and discharge, along with detailed therapy documentation. We trained models on data from adult patients admitted for a three-week post-acute knee rehabilitation program between 2018 and 2023.

We assessed the external validity of the models via validation in temporally and geographically distinct data collected in the training centre plus three additional centres from different regions in Austria, in 2024.

### 2.4 Participants

5,463 adult (≥18 years) patients undergoing the full three-week rehabilitation program after knee arthroplasty were initially available, 2,313 for training and 3,150 for testing (Figure 1). Patients were excluded if admission or discharge outcome assessments were missing. We further excluded patients for whom achieving a minimal clinically important difference (MCID) was mathematically impossible due to their extreme baseline values (i.e., ceiling effects; see ‘Outcome definitions and MCID operationalization’). This resulted in different datasets for each outcome with varying sample sizes (Figure 1). Excluded patients were compared with included patients on baseline characteristics using t-tests separately for each individual data set (Figure 1).

**Figure 1:**
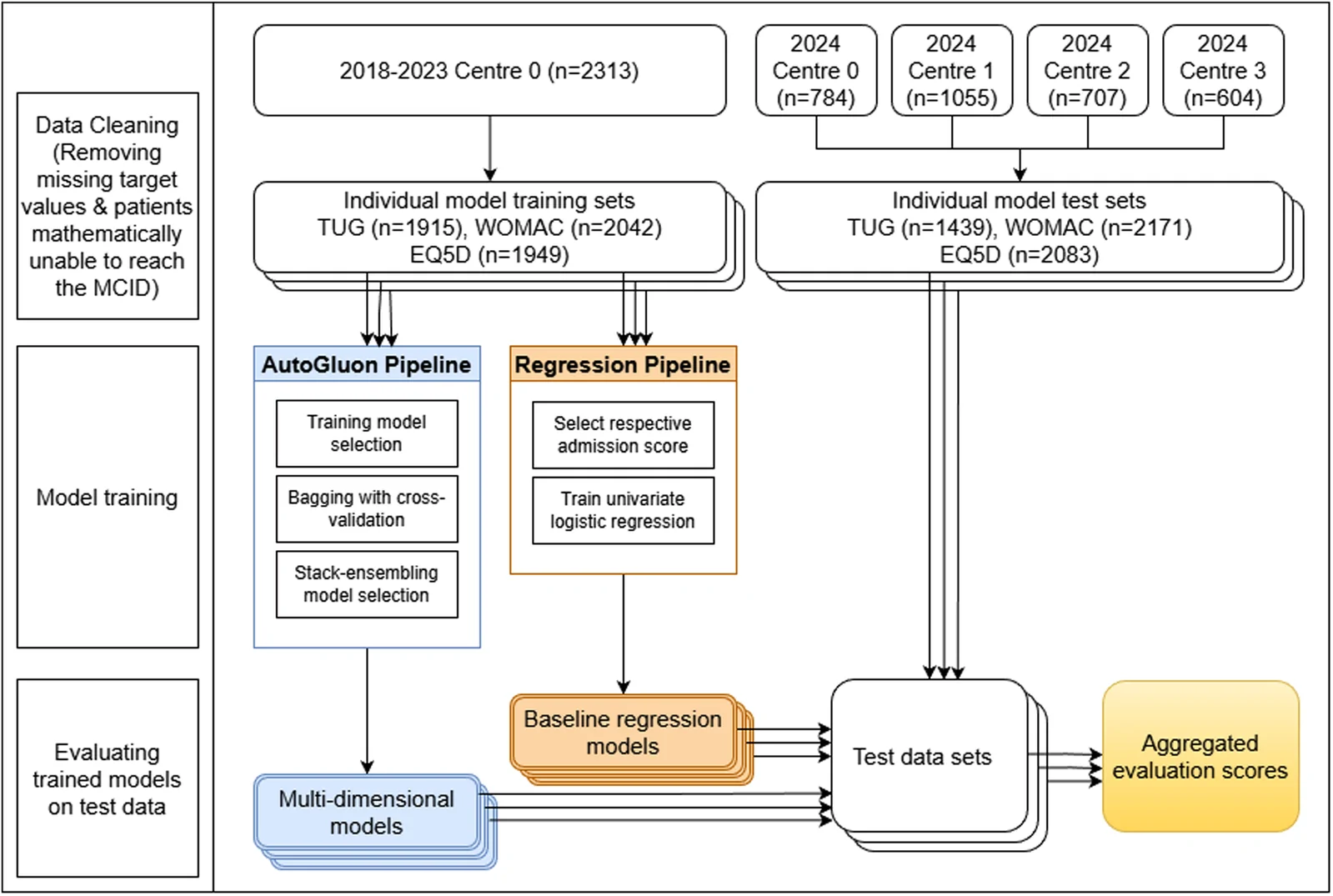
Study flowchart. MCID, Minimal Clinically Important Difference; TUG, Timed Up and Go test; WOMAC, Western Ontario and McMaster Universities Osteoarthritis Index; EQ5D, EQ-5D-5L-Index.

### 2.5 Outcome measures

Outcome measures were assessed at admission (baseline) and at discharge after three weeks of rehabilitation by trained clinical staff using standardized procedures.

#### 2.5.1 Patient-reported outcome measures

Health-related quality of life was measured using the EQ-5D-5L questionnaire, which assesses five dimensions (mobility, self-care, usual activities, pain/discomfort, and anxiety/depression) each on five levels (no, slight, moderate, severe, extreme problems) (38). Responses were converted into a single index score (EQ-5D-Index) using the German value sets, yielding values between 0 (equivalent to death) and 1 (perfect health). Knee-related pain, stiffness, and functional limitations were assessed using the Western Ontario and McMaster Universities Osteoarthritis Index (WOMAC). The questionnaire comprises 24 items: 5 pain items, 2 stiffness items, and 17 physical function items. Each item is rated on a 0–10 numeric rating scale, where 0 reflects the best and 10 the worst possible state. A total score is obtained by summing all items, yielding a 0–240 scale, with higher scores indicating worse symptoms. The WOMAC is a validated and widely used instrument to assess outcomes and satisfaction after hip and knee arthroplasty (15).

#### 2.5.2 Performance-based outcome

The Timed Up-and-Go (TUG) test is a performance-based measure of basic functional mobility and fall risk, originally described by Podsiadlo and Richardson (39). It has demonstrated good reliability, validity, and responsiveness across various populations, including orthopaedic and geriatric patients (39–44). After a standardized explanation, patients were instructed to rise from a standard armchair, walk three meters at a comfortable and safe speed, turn around, walk back to the chair, and sit down. The time from the command “go” until the patient's back contacted the chair was recorded in seconds using a stopwatch. Lower TUG times indicate better mobility.

### 2.6 Outcome definition and MCID operationalization

#### 2.6.1 Primary composite outcome

For each of the three outcome measures (TUG, EQ-5D-Index, WOMAC), we calculated change scores as the difference between discharge and admission values. Rehabilitation “success” and “non-response” were defined in terms of achieving a minimal clinically important difference (MCID) on these change scores. For each instrument, a binary outcome was defined:

- Responder: individual change ≥ instrument-specific MCID (i.e., improvement beyond MCID in the favourable direction).
- Non-responder: individual change < instrument-specific MCID.

#### 2.6.2 MCID derivation

MCID estimates are inherently context- and population-dependent rather than fixed properties of an outcome instrument (45). We therefore derived a study-specific distribution-based threshold from the training cohort rather than applying a published MCID obtained in a different population or setting. An anchor-based approach would have been preferable; however, as the study exclusively used routinely collected data, no global rating of change or comparable independent anchor was available. Based on a small effect size relative to the observed variability of within-person change scores (46, 47), the MCID for each instrument was defined as:

$$MCID_{\Delta }\;=0.2\;SD(\Delta )$$

Where SD (*Δ*) is the standard deviation of the observed change scores between admission and discharge. We selected 0.2 to represent a minimal/small, standardized change rather than the moderate change represented by 0.5 (46–48). Importantly, Woaye-Hune et al. (48) explicitly describe 0.2, one-third, and 0.5 x SD of the change score as distribution-based MCID approaches and found estimates based on 0.2 and one-third SD to correspond more closely to anchor-based estimates than more stringent distribution-based approaches. To assess the robustness of the findings to the MCID definition, all analyses were repeated using a more conservative alternative distribution-based threshold of 0.5 × SD of the admission scores. Based on the study data, this yielded the following MCID thresholds, shown in Table 1.

**Table 1:** Outcome distribution in the training sets.

Variable	Admission value^1^ [mean (SD)]	|Delta_Train_ (T1-T0)|^1^ [mean (SD)]	MCID^2^ (absolute change)	Non-responders^2^ (n, %)	MCID^3^ (admission)	Non-responders^3^ (n, %)
TUG (s)	11.99 (5.66)	2.79 (3.26)	0.65	355 (18.5)	2.83	856 (52.5)
WOMAC (0–240)	67.47 (41.49)	22.18 (29.48)	5.90	541 (26.5)	20.75	808 (44.5)
EQ-5D-Index (0–1)	0.83 (0.16)	0.05 (0.14)	0.03	838 (43.0)	0.08	985 (61.1)

TUG, Timed Up and Go Test; WOMAC, Western Ontario McMaster University Osteoarthritis Index; EQ-5D index, EuroQol 5-Dimensions Questionnaire Index; SD, standard deviation; T1-T0, discharge value minus admission value; MCID, minimal clinically important difference.

1 Based on the original training data.

2 Training set; MCIDs are derived from 0.2 x SD(Delta[Trainset]) to ensure the complete independence of test data.

3 Alternative training set; MCIDs are derived from 0.5 x SD(Admission[Trainset]) to ensure the complete independence of test data.

#### 2.6.3 Handling floor/ceiling and baseline dependence

MCID-based classification can be distorted by floor and ceiling effects and by baseline dependence, as patients with extreme good baseline scores are unable to achieve a given change for mathematical reasons (22, 49, 50). To avoid classifying such patients as “non-responders” or “responders” solely because of scale limits, we excluded individuals whose baseline scores implied an unattainable MCID.

In line with previous registry-based work in arthroplasty outcomes (19, 20), we applied the following rule: patients were excluded for a given outcome if the maximum possible change from their baseline score to the best (or worst) possible score on the scale was smaller than the instrument-specific MCID. For the TUG, we additionally imposed a pragmatic lower bound of 5 s at admission, based on normative reference data (51), acknowledging that the theoretical minimum of 0 s is physiologically impossible. This procedure resulted in the exclusion (for the respective outcome) of 34 patients for TUG, 63 for WOMAC, and 163 for the EQ-5D-Index. In the more conservative sensitivity analyses 356 (TUG), 299 (WOMAC), and 517 (EQ-5D-Index) patients were excluded, respectively.

### 2.7 Model development

#### 2.7.1 Predictor variables

For model development, we utilized all routinely collected baseline variables available in the registry. These included:

- Sociodemographic variables: age, sex.
- Clinical status at admission: baseline values of TUG, EQ-5D-Index, individual EQ-5D dimensions, and EQ-VAS (missing in two validation centres), WOMAC total score, Health Assessment Questionnaire (HAQ), Barthel index, Pain visual analogue scale, main diagnosis and Body Mass Index (BMI; missing in three validation centres)

Missing predictor variables (Supplementary Table S1) were not imputed and implicitly handled by the machine-learning algorithm (52). Outliers were not explicitly excluded or adjusted, as ensemble models are relatively robust to the influence of extreme values (53).

#### 2.7.2 Machine-learning algorithms (AutoML)

Model development was performed using AutoGluon (v1.4.0), an open-source automated machine learning (54) framework for tabular data (52). AutoGluon automates model selection, hyperparameter tuning, and ensembling across multiple algorithms, including gradient boosting, random forests, and neural networks, to enhance predictive performance. Trained models are combined through stacked ensembling to form a meta-model that integrates their predictive strengths. The “best quality” preset was applied, which employs eight-fold cross-validation: the training data are partitioned into eight folds, models are trained on seven folds and evaluated on the remaining fold, and the resulting out-of-fold predictions are concatenated to generate performance estimates.

#### 2.7.3 Baseline logistic regression models

As conventional benchmarks, we fitted univariate logistic regression models for each outcome definition using the respective admission value as the single predictor (e.g., baseline WOMAC predicting WOMAC non-response). These models represent the simplest plausible clinical decision rule based solely on baseline outcome status and allow comparison of the added value of multivariable machine-learning models.

### 2.8 Model performance and validation

Model performance was evaluated on held-out test data, collected in 2024, originating from three independent centres, providing geographical validation, and temporally independent data from the training centre (55, 56).

Discrimination was quantified by the area under the receiver operating characteristic curve (AUC), and Accuracy. For evaluation of classification (sensitivity, specificity, and F1 score), the decision threshold was set to the respective outcome prevalences observed in the training data (TUG: 0.185; WOMAC: 0.265; EQ-5D-Index: 0.430) and subsequently applied unchanged to the test set. To avoid reliance on a single potentially arbitrary *a priori* threshold, sensitivity and specificity were additionally evaluated across the full range of possible decision thresholds from 0 to 1, as recommended by Wynants et al. (57). Higher scores close to the maximum of 1 indicate better model performance for all the metrics above (58). Overall accuracy of probabilistic predictions was assessed with the Brier score (59), where lower values closer to 0 identify better models. Calibration was evaluated using the Integrated Calibration Index (ICI) (60), the calibration slope and calibration intercept. Good calibration is indicated by a low ICI, calibration slope close to 1 and calibration intercept around 0 (61).

To obtain 95% confidence intervals (CI) for performance metrics, we used non-parametric bootstrap resampling of the test set with 1,000 repetitions. For each bootstrap sample, the full set of metrics was recomputed, and percentile-based 95% CI were derived from the empirical distributions. Differences in AUC between models were compared using the non-parametric DeLong test (62). Model calibration and discrimination were further examined visually using confusion matrices and calibration plots (61).

Model interpretability was assessed using Shapley Additive exPlanations (SHAP) analysis based on the KernelExplainer (63). To ensure computational feasibility, SHAP values were estimated on a random subset of test samples (*n* = 50), using a representative background sample from the training data (*n* = 100).

Further, decision curve analyses (DCA) were performed to assess clinical utility across a range of threshold probabilities, reflecting divergent clinical preferences and potential follow-up opportunities (57, 64). Since subsequent downstream actions vary substantially, risk thresholds cannot be predefined.

## 3 Results

Outcome data from 5,463 patients was initial included, mean age 67.4 (SD = 12.3), 62.0% (2,799/4,511) female, mean BMI 29.4 (SD = 5.5, *n* = 3,020). Supplementary Table S1 displays the patient characteristics for each centre. The EQ-5D showed the highest prevalence of non-responders (48.5%), followed by WOMAC (27.6%), and TUG (20.7%). Rates of non-response varied significantly between the validation centres (TUG: Chi^2^ = 18.795, *p* < .001, range 13.0–27.0%; WOMAC: Chi^2^ = 14.993, *p* = .001, range 26.5–35.9%; EQ-5D: Chi^2^ = 35.008, *p* < .001, range 43.0–63.0%; Supplementary Table S1). Rates of missingness differed between centres for each target (Supplementary Table S1); TUG 17.2%–79.7%, WOMAC 11.7%–43.3%, and EQ-5D-Index 15.7%–45.8%. Supplementary Table S2 shows the sensitivity analysis comparing included and excluded patients on baseline characteristics, showing a slightly worse health state in excluded patients in the test data and the reverse effect in the training data.

### 3.1 Model evaluation

Descriptively, the multivariate ensemble models outperformed the discriminatory ability (AUC) of the baseline regressions in all targets, with a significant difference observed for the PROMs EQ-5D-Index (*p* = .001) and WOMAC (*p* = .008), but not for the performance-based TUG (*p* = .638), see Supplementary Figure S1 for a comparison of receiver operating characteristics (ROC) curve.

Model evaluation based on the pooled validation (see Supplementary Table S3 for individual centres) yielded good discriminatory performance (Table 2). Highest AUCs could be found for TUG (0.771, 95% CI [0.742, 0.799]), followed by EQ-5D-Index (0.770, 95% CI [0.749, 0.789]) and WOMAC (0.704, 95% CI [0.679, 0.730]). Normalized confusion matrices with cut-offs based on prevalence are shown in Supplementary Figure S2 and an assessment of sensitivity and specificity shifts across multiple operating thresholds in Supplementary Figure S3.

**Table 2:** Pooled model evaluation results.

Target	TUG		WOMAC		EQ-5D-Index	
Algorithm	AG	LR	AG	LR	AG	LR
AUC 95%-CI	0.771 [0.742, 0.799]	0.769 [0.740, 0.798]	0.704 [0.679, 0.730]	0.689 [0.663, 0.715]	0.770 [0.749, 0.789]	0.748 [0.727, 0.768]
DeLong test (*p*-value)^1^	0.638		**0.008**		**0.001**	
Brier score 95%-CI	0.152 [0.139, 0.165]	0.150 [0.138, 0.163]	0.180 [0.171, 0.189]	0.186 [0.178, 0.195]	0.237 [0.228, 0.245]	0.223 [0.216, 0.230]
Accuracy 95%-CI^2^	0.730 [0.705, 0.751]	0.714 [0.690, 0.737]	0.663 [0.644, 0.683]	0.577 [0.556, 0.599]	0.655 [0.636, 0.675]	0.675 [0.655, 0.695]
F1 95%-CI^2^	0.540 [0.500, 0.577]	0.533 [0.492, 0.571]	0.519 [0.489, 0.549]	0.490 [0.461, 0.519]	0.593 [0.567, 0.619]	0.634 [0.608, 0.658]
Sensitivity 95%-CI^2^	0.673 [0.625, 0.721]	0.694 [0.648, 0.741]	0.634 [0.598, 0.674]	0.709 [0.672, 0.746]	0.469 [0.441, 0.497]	0.524 [0.495, 0.552]
Specificity 95%-CI^2^	0.747 [0.719, 0.774]	0.720 [0.694, 0.745]	0.675 [0.652, 0.698]	0.525 [0.498, 0.549]	0.870 [0.850, 0.892]	0.850 [0.829, 0.872]
ICI 95%-CI	0.073 [0.055, 0.090]	0.060 [0.044, 0.077]	0.042 [0.032, 0.054]	0.063 [0.050, 0.078]	0.192 [0.172, 0.212]	0.155 [0.136, 0.175]
Calibration intercept 95%-CI	0.356 [0.126, 0.580]	−0.018 [−0.234, 0.201]	0.089 [−0.061, 0.242]	−0.085 [−0.244, 0.072]	1.142 [0.990, 1.309]	0.779 [0.661, 0.912]
Calibration slope 95%-CI	0.865 [0.747, 0.994]	0.645 [0.516, 0.794]	0.956 [0.832, 1.088]	0.867 [0.710, 1.035]	1.289 [1.157, 1.433]	1.162 [0.979, 1.350]

LR, baseline logistic regression; AG, multivariable ensemble AutoGluon model; AUC, area under the receiver operating characteristics curve; CI, confidence interval; ICI, integrated calibration index; TUG, Timed Up and Go Test; WOMAC, Western Ontario McMaster University Osteoarthritis Index; EQ-5D-Index, EuroQol 5-Dimensions Questionnaire Index.

Interpretation: AUC, Accuracy, F1: higher is better (maximum 1.0); Brier score, ICI: lower is better (minimum 0.0); Calibration intercept: ideal = 0; Calibration slope: ideal = 1.

1 A *p*-value < 0.05 indicates a significant difference between the AUCs of the multivariable ensemble and baseline logistic regression model.

2 Decision threshold set to outcome prevalences in the training data: TUG: 0.185; WOMAC: 0.265; EQ-5D-Index: 0.430.

Bold values indicate a significant difference between the AUCs of the multivariable ensemble and baseline logistic regression model.

Calibration, evaluated based on the calibration plots (Supplementary Figure S4), was acceptable with curves lying minimally above the diagonal for TUG and WOMAC but only covered values in the lower probability range. For the EQ-5D, however, deviations from the diagonal were greatest at low predicted probabilities, also showing an underestimation of risks. Calibration slopes indicate accurate risk predictions for WOMAC (0.956, 95% CI [0.832, 1.088]), and a spread of estimated risks for TUG (0.865, 95% CI [0.747, 0.994]; overly extreme), and EQ-5D-Index (1.289, 95% CI [1.157, 1.433]; too moderate). Similarly, calibration intercepts show accurate estimations for WOMAC (0.089, 95% CI [−0.061, 0.242]) and underestimation for TUG (0.356, 95% CI [0.126, 0.580]) and EQ-5D-Index (1.142, 95% CI [0.990, 1.309]).

When model performance was examined across individual centres (Supplementary Table S3), centre 2 consistently demonstrated the highest performance (AUCs: TUG (*n* = 230) 0.882, 95% CI [0.813, 0.941]; WOMAC (*n* = 401) 0.748, 95% CI [0.690, 0.803]; EQ-5D-Index (*n* = 417) 0.816, 95% CI [0.774, 0.854]), including outperforming the temporal validation of the training centre, with no clear association with centre-specific event rates, but based on the lowest total number of events (Supplementary Table S1).

Decision curve analyses (Supplementary Figure S5) revealed a superior clinical utility (higher net benefit) of the ensemble models compared to the “treat all” scenario for risk threshold between 0.1 and 0.6 (TUG), 0.2–0.6 (WOMAC), and 0.5–0.8 (EQ-5D-Index). The baseline regression showed similar net benefits for TUG and WOMAC, but superior net benefit between 0.2 and 0.5 for the EQ-5D-Index.

Sensitivity analyses with the alternative, admission-based MCID definition, are provided in Supplementary Table S4. They largely follow the same patterns with slightly improved discrimination.

### 3.2 Predictor importance

All 16 predictors described in section 2.7.1 were included in the models. SHAP analyses revealed the respective admission values to be the most important predictors within the single target models with a substantial margin over the next-best predictors (Figure 2). In all cases, better values resulted in a higher likelihood of not achieving a MCID.

**Figure 2:**
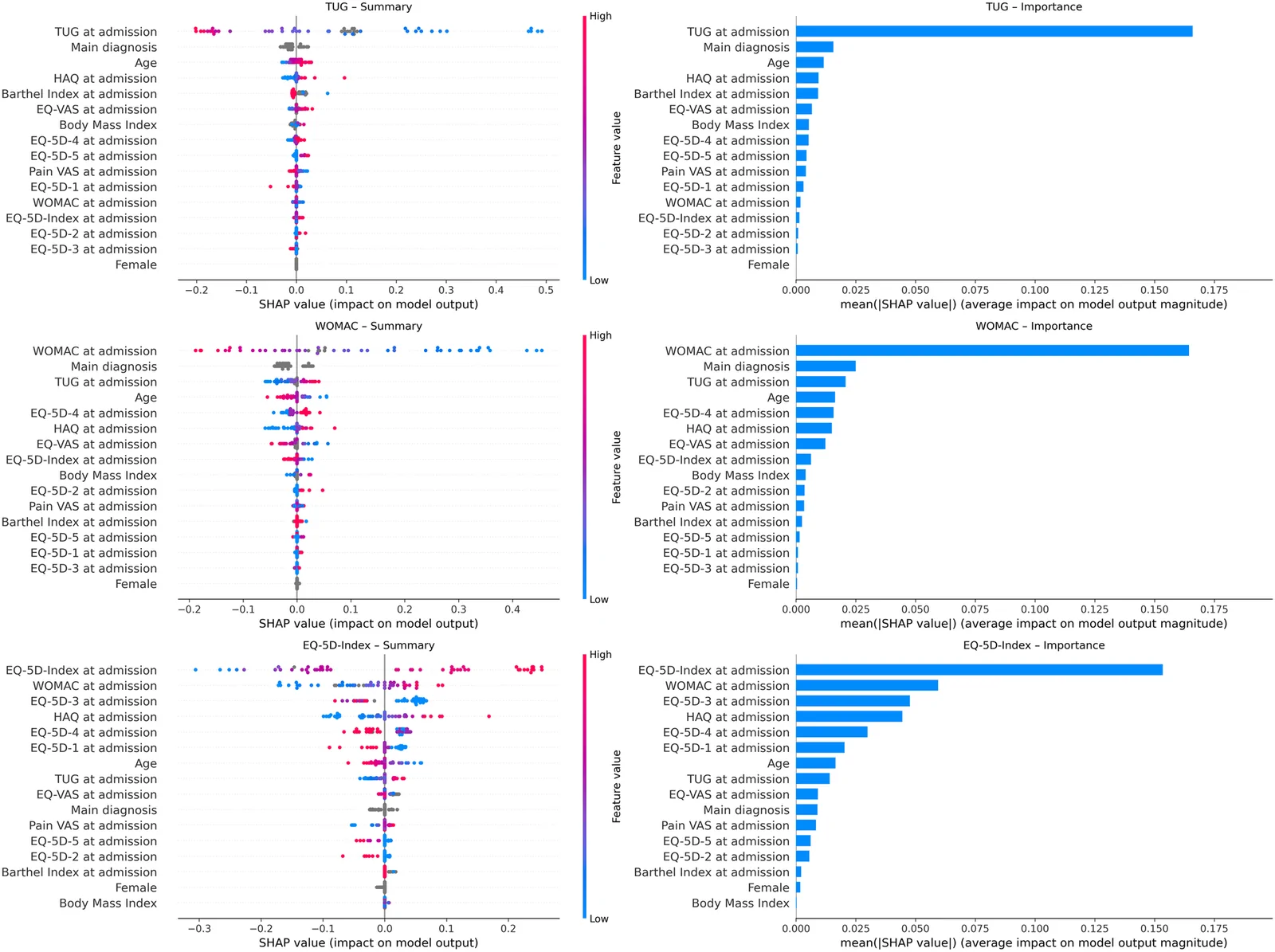
Feature importance (SHAP analyses). TUG, Timed Up and Go Test; WOMAC, Western Ontario McMaster University Osteoarthritis Index; EQ-5D Index, EuroQol 5-Dimensions Questionnaire Index; EQ-VAS, EuroQol Visual Analogue Scale; EQ-5D-5L-1, EuroQol 5-Dimensions Questionnaire mobility dimension; EQ-5D-5L-2, EuroQol 5-Dimensions Questionnaire self-care dimension; EQ-5D-5L-3, EuroQol 5-Dimensions Questionnaire usual activities dimension; EQ-5D-5L-4, EuroQol 5-Dimensions Questionnaire pain dimension; EQ-5D-5L-5, EuroQol 5-Dimensions Questionnaire anxiety/depression dimension; HAQ, Health Assessment Questionnaire; Pain VAS, Pain Visual Analogue Scale; Variables are sorted in descending order of importance. In the summary plots (left side), red dots indicate high variable values (for TUG, WOMAC, Pain VAS, individual EQ-5D domains, and HAQ lower values indicate a better score) and positive *x*-axis values imply an increased probability of not reaching the MCID (TUG, WOMAC, and EQ-5D). The importance plots (right side) show the mean absolute value of each predictor.

For the TUG model, this was followed by the main diagnosis, higher age, and a higher degree of limitations in daily life (HAQ), which led to an increase in the probability to not achieve an MCID.

WOMAC models were further driven by the main diagnosis, slower TUG admission values and lower age. Whereas the EQ-5D model was mainly influenced by PROMs (WOMAC, EQ-5D domains, HAQ). Interestingly, sex was not among the most important predictors for any outcome.

## 4 Discussion

Machine learning models were able to predict non-response across multiple rehabilitation outcomes with discriminative performance of AUC > 0.7 and moderate calibration in independent validation data. Compared with a baseline-only model using admission scores alone, ML provided clear added predictive value for the patient-reported WOMAC. The EQ-5D showed superior discrimination, but poor calibration, and no additional benefit was observed for the performance-based TUG. Non-response was strongly dependent on applied outcome assessments, reflecting varying responsiveness of functional and patient-reported measures, with substantially varying proportions of non-responders across outcomes. Feature importance analyses showed that predicted non-response was strongly driven by better baseline functional status.

### 4.1 Model performance in context

Our observed performance patterns were consistent with the growing body of literature on MCID-based PRO prediction after joint replacement and rehabilitation (32). Huber et al. (28) demonstrated high predictive performance for EQ-VAS and Oxford scores in a large British cohort of TKA patients, similarly identifying baseline PROMs as the most influential predictors, but without reporting calibration. Comparable AUC ranges, meaning the models showed similar ability to distinguish between responders and non-responders, and a limited incremental benefit of ML over conventional regression have also been reported (19, 65). Current literature comparing ML-based prediction models for patient-reported and performance-based outcomes is rare. In a TKA patient sample superior classification (weighted F1-score) for TUG compared with WOMAC models was reported, although based on a three-classed grading scheme (26).

Outcomes defined by change scores are inherently influenced by patients’ baseline status, shaping both their interpretation and predictive modelling (66). This strong dependence on baseline status can be understood not only as a clinical phenomenon but also as a structural property of MCID-based outcome definitions. When response is operationalized as achieving a minimum change threshold, the achievable improvement is inherently constrained by the baseline level due to scale limits and regression-to-the-mean effects (67). This coupling reduces the scope for additional predictors to contribute independent information, particularly when only routine clinical variables are available. In this context, ensemble models may offer limited advantages unless non-linear interactions or richer predictor domains, such as psychosocial factors, pain coping, or comorbidities are incorporated, as shown in related work (10, 68, 69). The stronger performance gains of the ensemble over the baseline regression observed for WOMAC and EQ-5D compared with TUG further suggest that PROMs may benefit more from such extended predictor sets, given their broader conceptual scope and susceptibility to systematic measurement error (specifically common method bias 70).

### 4.2 Clinical implications

From a clinical perspective, these results help refine expectations regarding the role of ML in inpatient TKA rehabilitation. Our findings suggest that outcome-specific decision support is more realistic than a single global prediction model, with functional outcomes (TUG & WOMAC) being more amenable to prediction using routinely collected data, whereas accurate prediction of global HRQoL may require additional predictors, for example psychosocial, behavioural or environmental factors.

At the same time, the higher non-response rates observed for EQ-5D imply that short-term rehabilitation may have limited impact on global quality of life, reinforcing the need for complementary strategies beyond physical training alone (e.g., psychological support). Importantly, the tendency for fitter patients to be classified as non-responders underscores the relevance of ceiling effects and the importance of expectation management, rather than indicating treatment failure *per se*.

For clinical implementation, case-specific decision thresholds will be needed to translate predicted probabilities into clinically meaningful low- and high-risk classifications. Such thresholds should be established on medical rather than purely statistical grounds, taking into account the intended clinical decision and the relative consequences of false-positive and false-negative classifications (57). However, the decision curve analyses presented here already yield a range of thresholds with potential clinical utility. In the context of rehabilitation, lower thresholds favouring sensitivity may be appropriate when identifying patients who could benefit from additional supportive interventions, particularly where false-positive classifications are unlikely to result in substantial harm.

The current models provide a structured foundation for future work aiming at integrating additional predictors, refining outcome definitions (such as baseline-adapted MCIDs), and translating probabilistic risk estimates into actionable rehabilitation pathways.

### 4.3 Strength and limitations

This study is based on real-world rehabilitation data from a routine clinical setting, including standardized admission and discharge assessments collected between multiple years (2018–2024) and clinically relevant outcomes (PROMs and performance-based measures). The models were temporally and geographically validated in independent data with significantly varying outcome prevalences, strengthening robustness and generalizability. A step often lacking in clinical prediction modelling studies, which might limit their broader application in clinical settings (32, 71). This should also be considered when comparing the performance of the models presented here with studies reporting performance based solely on internal validation, which generally yield more optimistic estimates (72, 73). A key strength is the explicit focus on non-response, a clinically meaningful target that supports individualized therapy steering. The approach enables early identification of potential non-responders, informing treatment intensification or modification, expectation management, and follow-up planning. At the same time, non-response predictions must be applied cautiously to avoid labelling effects or self-fulfilling prophecies, such as reduced resource allocation for patients with a poor predicted prognosis and should therefore serve as a supportive information source rather than a gatekeeping instrument, with attention to fairness across vulnerable patient subgroups.

Nevertheless, this study contains some limitations. First, removing cases with missing target variables without imputation may introduce systematic selection bias, as missing outcomes could be more frequent in complex cases, potentially inflating performance. Differing missingness mechanisms across validation datasets may also affect calibration and lead to performance drift and affect between centre comparisons. Second, non-response was defined pragmatically using a distribution-based MCID and dichotomization, which is context-specific and may substantially influence non-responder prevalence and predictability. The selection and characteristics of the indicators, particularly the definition of the endpoint (i.e., a reliable and valid gold standard), have a significant impact on predictive models. Importantly, the sensitivity analysis also relied on a distribution-based MCID and therefore does not overcome this limitation, although it demonstrated robustness to more conservative threshold definition. Third, the Austrian study population limits generalizability, and external validation in international settings is needed. Further, SHAP-based variable importance should be interpreted with some caution, as SHAP values were estimated using a random subsample due to computational constraints. However, this limitation is partly mitigated by the clear dominance of baseline status, which consistently contributed substantially more to the model predictions than any other predictor. Consequently, while the precise ordering of the remaining, comparatively marginal predictors may be less stable, this is unlikely to affect the main interpretation of the variable importance analysis. And finally, clinical implementation is not feasible yet. This reflects a broader systemic challenge in translational implementation, whereby HRQoL prediction models frequently fail to advance beyond the proof-of-concept stage due to a combination of technical, operational, and contextual barriers (74). As described by Spencer et al. (75), both general machine-learning deployment limitations and challenges specific to patient-reported outcomes contribute to what they term a “leaky pipeline” between model development and bedside application. In addition, adherence to regulatory frameworks such as the EU AI Act requires not only rigorous prospective validation in real-world settings, but also clear alignment with ethical principles, safety requirements, and stakeholder-engagement standards.

## 5 Conclusions

Machine learning shows promise for predicting outcomes in patients undergoing TKA rehabilitation. Routinely available patient characteristics can be leveraged to identify patients who are likely to require a specialized therapeutic approach to substantially improve functional outcomes and HRQoL. While practical implementation remains challenging, ML-based decision support may still offer meaningful clinical value by providing information for personalized, patient-centered expectation management. The current models offer a structured foundation for future work aiming at integrating additional predictors, refining outcome definitions (such as baseline-adapted MCIDs), and translating probabilistic risk estimates into actionable rehabilitation pathways.

## Data Availability

The datasets generated and analysed in the current study are not publicly available due to data protection regulations. Requests to access the datasets should be directed to the corresponding author/s.
