# Supplementary material for: Development and validation of machine learning models for predicting rehabilitation non-response after knee arthroplasty
Source: Front Rehabil Sci. 2026 Sep 18;7:1930836. doi: 10.3389/fresc.2026.1930836 (PMC13630954; doi:10.3389/fresc.2026.1930836)
Supplement: Supplementary file 1 [file Datasheet1.docx]

## Table S1: Patient characteristics and outcome distribution.

| Variables | Overall | Train (Centre 0) | Test (Centre 0) | Test (Centre 1) | Test (Centre 2) | Test (Centre 3) |
| --- | --- | --- | --- | --- | --- | --- |
| n | 5463 | 2313 | 784 | 1055 | 707 | 604 |
| Sex (n, %) |  |  |  |  |  |  |
| Male^1^ | 1712 (38.0) | 504 (37.0) | 327 (41.7) | 355 (33.6) | 278 (39.3) | 248 (41.1) |
| Female^1^ | 2799 (62.0) | 857 (63.0) | 457 (58.3) | 700 (66.4) | 429 (60.7) | 356 (58.9) |
| Missing | 952 (17.4) | 952 (41.2) | 0 (0.0) | 0 (0.0) | 0 (0.0) | 0 (0.0) |
| Age (mean (SD)) | 67.36 (12.28) | 65.45 (12.95) | 68.02 (12.25) | 68.66 (11.93) | 69.74 (10.52) | 68.80 (11.14) |
| Missing (n, %) | 0 (0.0) | 0 (0.0) | 0 (0.0) | 0 (0.0) | 0 (0.0) | 0 (0.0) |
| BMI (mean (SD)) | 29.40 (5.46) | 29.18 (5.52) | Missing | Missing | 29.93 (5.24) | Missing |
| Missing (n, %) | 3411 (62.4) | 844 (36.5) | 784 (100.0) | 1055 (100.0) | 124 (17.5) | 604 (100.0) |
|  |  |  |  |  |  |  |
| EQ-VAS at admission (mean (SD)) | 65.28 (19.15) | 65.61 (18.62) | 65.68 (20.26) | Missing | 63.71 (19.54) | Missing |
| Missing (n, %) | 1882 (34.4) | 120 (5.2) | 35 (4.5) | 1055 (100.0) | 68 (9.6) | 604 (100.0) |
| EQ-5D-Index at admission (mean (SD)) | 0.81 (0.16) | 0.83 (0.16) | 0.82 (0.13) | 0.78 (0.16) | 0.76 (0.17) | 0.84 (0.13) |
| Missing (n, %) | 370 (6.8) | 119 (5.1) | 38 (4.8) | 121 (11.5) | 68 (9.6) | 24 (4.0) |
| EQ-5D-5L-1 at admission (mean (SD)) | 1.95 (0.95) | 1.90 (0.94) | 1.89 (0.92) | 2.10 (0.97) | 2.16 (1.01) | 1.73 (0.87) |
| Missing (n, %) | 358 (6.6) | 119 (5.1) | 35 (4.5) | 114 (10.8) | 68 (9.6) | 22 (3.6) |
| EQ-5D-5L-2 at admission (mean (SD)) | 1.23 (0.62) | 1.25 (0.67) | 1.20 (0.55) | 1.20 (0.55) | 1.37 (0.71) | 1.14 (0.51) |
| Missing (n, %) | 358 (6.6) | 119 (5.1) | 35 (4.5) | 114 (10.8) | 68 (9.6) | 22 (3.6) |
| EQ-5D-5L-3 at admission (mean (SD)) | 1.82 (1.00) | 1.84 (1.00) | 1.73 (0.94) | 1.89 (0.96) | 2.07 (1.13) | 1.50 (0.84) |
| Missing (n, %) | 357 (6.5) | 119 (5.1) | 34 (4.3) | 114 (10.8) | 68 (9.6) | 22 (3.6) |
| EQ-5D-5L-4 at admission (mean (SD)) | 2.47 (0.82) | 2.43 (0.79) | 2.33 (0.80) | 2.62 (0.85) | 2.66 (0.83) | 2.30 (0.82) |
| Missing (n, %) | 358 (6.6) | 119 (5.1) | 35 (4.5) | 114 (10.8) | 68 (9.6) | 22 (3.6) |
| EQ-5D-5L-5 at admission (mean (SD)) | 1.37 (0.71) | 1.31 (0.64) | 1.32 (0.66) | 1.51 (0.84) | 1.47 (0.78) | 1.29 (0.65) |
| Missing (n, %) | 357 (6.5) | 119 (5.1) | 34 (4.3) | 114 (10.8) | 68 (9.6) | 22 (3.6) |
| HAQ at admission (mean (SD)) | 0.36 (0.34) | 0.34 (0.33) | 0.32 (0.32) | 0.39 (0.36) | 0.49 (0.38) | 0.27 (0.31) |
| Missing (n, %) | 363 (6.6) | 120 (5.2) | 35 (4.5) | 114 (10.8) | 71 (10.0) | 23 (3.8) |
| Pain VAS at admission (mean (SD)) | 4.04 (2.09) | 3.97 (2.02) | 3.80 (2.04) | 4.47 (2.12) | 4.56 (2.16) | 3.38 (2.01) |
| Missing (n, %) | 363 (6.6) | 119 (5.1) | 35 (4.5) | 114 (10.8) | 72 (10.2) | 23 (3.8) |
| WOMAC at admission (mean (SD)) | 65.88 (42.70) | 67.47 (41.49) | 67.06 (42.17) | 73.34 (45.38) | 66.28 (44.69) | 46.10 (35.75) |
| Missing (n, %) | 620 (11.3) | 120 (5.2) | 71 (9.1) | 157 (14.9) | 235 (33.2) | 37 (6.1) |
| Barthel at admission (mean (SD)) | 98.57 (3.93) | 98.35 (4.31) | 99.26 (2.77) | 98.93 (3.07) | 97.47 (4.81) | 99.62 (2.11) |
| Missing (n, %) | 1557 (28.5) | 315 (13.6) | 97 (12.4) | 554 (52.5) | 249 (35.2) | 342 (56.6) |
| TUG at admission (mean (SD)) | 12.01 (7.11) | 11.99 (5.66) | 11.46 (5.14) | 11.89 (12.71) | 14.14 (6.54) | 11.60 (4.61) |
| Missing (n, %) | 1150 (21.1) | 140 (6.1) | 140 (17.9) | 406 (38.5) | 387 (54.7) | 77 (12.7) |
|  |  |  |  |  |  |  |
| **Targets** |  |  |  |  |  |  |
| TUG (n, %) |  |  |  |  |  |  |
| Non-Responder^1^ | 694 (20.7) | 355 (18.5) | 144 (27.0) | 57 (26.6) | 30 (13.0) | 108 (23.4) |
| Missing | 2109 (38.61) | 398 (17.21) | 251 (32.02) | 841 (79.72) | 477 (67.47) | 142 (23.51) |
| WOMAC (n, %) |  |  |  |  |  |  |
| Non-Responder^1^ | 1163 (27.6) | 541 (26.5) | 174 (26.6) | 170 (26.5) | 108 (26.9) | 170 (35.9) |
| Missing | 1250 (22.88) | 271 (11.72) | 129 (16.45) | 414 (39.24) | 306 (43.28) | 130 (21.52) |
| EQ-5D (n, %) |  |  |  |  |  |  |
| Non-Responder^1^ | 1954 (48.5) | 838 (43.0) | 348 (55.8) | 287 (50.2) | 185 (44.4) | 296 (63.0) |
| Missing | 1431 (26.19) | 364 (15.74) | 160 (20.41) | 483 (45.78) | 290 (41.02) | 134 (22.19) |

*Notes: ^1^ only valid percentages are shown;*

*BMI: Body Mass Index; EQ-VAS: EuroQol Visual Analogue Scale; EQ-5D Index: EuroQol 5-Dimensions Questionnaire Index; EQ-5D-5L-1: EuroQol 5-Dimensions Questionnaire mobility dimension; EQ-5D-5L-2: EuroQol 5-Dimensions Questionnaire self-care dimension; EQ-5D-5L-3: EuroQol 5-Dimensions Questionnaire usual activities dimension; EQ-5D-5L-4: EuroQol 5-Dimensions Questionnaire pain dimension; EQ-5D-5L-5: EuroQol 5-Dimensions Questionnaire anxiety/depression dimension; VAS: Visual Analogue Scale; TUG: Timed Up and Go Test; WOMAC: Western Ontario McMaster University Osteoarthritis Index;*

## Table S2: Comparison of included and excluded patients on baseline characteristics in each individual data set.

| **Dataset** | **TUG(train)** | | | **WOMAC(train)** | | | **EQ-5D-Index(train)** | | | **TUG(test)** | | | **WOMAC(test)** | | | **EQ-5D-Index(test)** | | |
| --- | --- | --- | --- | --- | --- | --- | --- | --- | --- | --- | --- | --- | --- | --- | --- | --- | --- | --- |
| Variables | Excluded | Included | p-value | Excluded | Included | p-value | Excluded | Included | p-value | Excluded | Included | p-value | Excluded | Included | p-value | Excluded | Included | p-value |
| n | 398 | 1915 |  | 271 | 2042 |  | 364 | 1949 |  | 1711 | 1439 |  | 979 | 2171 |  | 1067 | 2083 |  |
| Female = 1 ´(n (%)) | 178 (62.2) | 679 (63.2) | .827 | 142 (65.7) | 715 (62.4) | .399 | 152 (56.3) | 705 (64.6) | **.014** | 1075 (62.8) | 867 (60.3) | .148 | 636 (65.0) | 1306 (60.2) | **.011** | 679 (63.6) | 1263 (60.6) | .109 |
| Age (mean (SD)) | 64.29 (14.61) | 65.69 (12.57) | **.049** | 69.20 (14.16) | 64.95 (12.70) | **<.001** | 67.60 (14.15) | 65.05 (12.67) | **.001** | 68.68 (11.83) | 68.87 (11.26) | .651 | 70.04 (12.15) | 68.20 (11.26) | **<.001** | 69.90 (11.82) | 68.19 (11.40) | **<.001** |
| BMI (mean (SD)) | 28.93 (5.93) | 29.22 (5.46) | .484 | 29.10 (4.85) | 29.19 (5.58) | .868 | 29.23 (5.02) | 29.17 (5.59) | .906 | 29.82 (5.53) | 30.13 (4.68) | .497 | 30.11 (5.00) | 29.83 (5.39) | .525 | 29.94 (5.02) | 29.93 (5.37) | .986 |
| EQ-VAS at  admission (mean (SD)) | 66.03 (19.31) | 65.52 (18.48) | .631 | 70.95 (20.48) | 65.21 (18.42) | **<.001** | 72.78 (20.49) | 64.71 (18.18) | **<.001** | 63.23 (20.21) | 66.04 (19.64) | **.009** | 65.80 (20.75) | 64.45 (19.68) | .285 | 66.48 (21.41) | 64.20 (19.41) | .066 |
| EQ-5D-Index at  admission (mean (SD)) | 0.83 (0.17) | 0.83 (0.15) | .813 | 0.89 (0.12) | 0.82 (0.16) | **<.001** | 0.95 (0.10) | 0.81 (0.16) | **<.001** | 0.79 (0.16) | 0.81 (0.14) | **<.001** | 0.78 (0.18) | 0.80 (0.14) | **.001** | 0.82 (0.18) | 0.79 (0.14) | **<.001** |
| EQ-5D-5L-1 at  admission (mean (SD)) | 1.87 (0.91) | 1.90 (0.95) | .479 | 1.60 (0.88) | 1.92 (0.94) | **<.001** | 1.40 (0.69) | 1.96 (0.95) | **<.001** | 2.05 (0.97) | 1.92 (0.94) | **<.001** | 2.04 (1.01) | 1.97 (0.94) | .063 | 1.87 (1.00) | 2.03 (0.94) | **<.001** |
| EQ-5D-5L-2 at  admission (mean (SD)) | 1.24 (0.68) | 1.25 (0.67) | .823 | 1.14 (0.51) | 1.25 (0.68) | **.0380** | 1.05 (0.28) | 1.27 (0.70) | **<.001** | 1.25 (0.61) | 1.21 (0.56) | .057 | 1.27 (0.64) | 1.21 (0.57) | **.009** | 1.23 (0.59) | 1.23 (0.59) | .966 |
| EQ-5D-5L-3 at  admission (mean (SD)) | 1.81 (0.97) | 1.84 (1.01) | .592 | 1.52 (0.89) | 1.86 (1.01) | **<.001** | 1.27 (0.68) | 1.91 (1.01) | **<.001** | 1.88 (1.00) | 1.74 (0.97) | **<.001** | 1.88 (1.06) | 1.79 (0.97) | **.032** | 1.74 (0.98) | 1.84 (0.99) | **.012** |
| EQ-5D-5L-4 at  admission (mean (SD)) | 2.41 (0.84) | 2.43 (0.78) | .640 | 1.92 (0.83) | 2.47 (0.77) | **<.001** | 1.46 (0.76) | 2.55 (0.70) | **<.001** | 2.55 (0.86) | 2.43 (0.81) | **<.001** | 2.50 (0.95) | 2.49 (0.80) | .781 | 2.25 (1.03) | 2.59 (0.73) | **<.001** |
| EQ-5D-5L-5 at  admission (mean (SD)) | 1.33 (0.66) | 1.30 (0.64) | .493 | 1.28 (0.68) | 1.31 (0.64) | .576 | 1.11 (0.39) | 1.33 (0.66) | **<.001** | 1.46 (0.79) | 1.36 (0.71) | **<.001** | 1.51 (0.86) | 1.37 (0.71) | **<.001** | 1.43 (0.80) | 1.40 (0.74) | .397 |
| HAQ at admission  (mean (SD)) | 0.31 (0.31) | 0.35 (0.33) | **.035** | 0.21 (0.29) | 0.35 (0.33) | **<.001** | 0.18 (0.26) | 0.36 (0.33) | **<.001** | 0.39 (0.37) | 0.34 (0.33) | **<.001** | 0.41 (0.41) | 0.36 (0.33) | **<.001** | 0.37 (0.39) | 0.37 (0.34) | .742 |
| Pain VAS at  admission (mean (SD)) | 3.93 (2.08) | 3.98 (2.01) | .713 | 2.61 (2.31) | 4.07 (1.96) | **<.001** | 2.30 (2.07) | 4.18 (1.91) | **<.001** | 4.23 (2.15) | 3.97 (2.12) | **.001** | 4.05 (2.41) | 4.12 (2.04) | .447 | 3.87 (2.34) | 4.19 (2.05) | **<.001** |
| WOMAC at  admission (mean (SD)) | 63.73 (41.41) | 68.23 (41.48) | .056 | 39.61 (44.66) | 69.53 (40.51) | **<.001** | 39.68 (39.47) | 70.98 (40.41) | **<.001** | 67.43 (45.69) | 61.59 (41.23) | **.001** | 61.63 (53.33) | 65.21 (41.19) | .104 | 62.44 (49.49) | 65.36 (41.21) | .125 |
| Barthel at admission  (mean (SD)) | 98.35 (4.19) | 98.35 (4.34) | .976 | 97.55 (5.39) | 98.47 (4.13) | **.002** | 98.06 (4.85) | 98.41 (4.20) | .184 | 98.47 (3.92) | 99.17 (2.80) | **<.001** | 98.27 (4.60) | 98.93 (3.09) | **.001** | 98.44 (4.21) | 98.92 (3.13) | **.008** |
| TUG at admission  (mean (SD)) | 10.60 (5.10) | 12.17 (5.71) | **<.001** | 13.19 (7.48) | 11.83 (5.36) | **<.001** | 12.18 (6.86) | 11.95 (5.41) | .485 | 12.21 (12.63) | 11.94 (5.03) | .478 | 13.31 (13.99) | 11.60 (5.21) | **<.001** | 12.76 (13.28) | 11.74 (5.28) | **.012** |

*Notes: Patients were excluded if admission or discharge outcome assessments were missing; p-values < 0.05 in bold*

*BMI: Body Mass Index; EQ-VAS: EuroQol Visual Analogue Scale; EQ-5D Index: EuroQol 5-Dimensions Questionnaire Index; EQ-5D-5L-1: EuroQol 5-Dimensions Questionnaire mobility dimension; EQ-5D-5L-2: EuroQol 5-Dimensions Questionnaire self-care dimension; EQ-5D-5L-3: EuroQol 5-Dimensions Questionnaire usual activities dimension; EQ-5D-5L-4: EuroQol 5-Dimensions Questionnaire pain dimension; EQ-5D-5L-5: EuroQol 5-Dimensions Questionnaire anxiety/depression dimension; VAS: Visual Analogue Scale; TUG: Timed Up and Go Test; WOMAC: Western Ontario McMaster University Osteoarthritis Index*

## Table S3: Model evaluation results multivariable ensemble models per centre.

| **Target** | **Dataset** | **AUC** | **95% CI** | **Brier** | **95% CI** | **Accuracy^1^ 95% CI** | | **F1^1^** | **95% CI** | **Sensitivity^1^ 95% CI** | | **Specificity^1^ 95% CI** | | **ICI** | **95% CI** | **Intercept 95% CI** | | **Slope** | **95% CI** |
| --- | --- | --- | --- | --- | --- | --- | --- | --- | --- | --- | --- | --- | --- | --- | --- | --- | --- | --- | --- |
| TUG | Pooled | 0.771 | [0.742, 0.799] | 0.152 | [0.139, 0.165] | 0.730 | [0.705, 0.751] | 0.540 | [0.500, 0.577] | 0.673 | [0.625, 0.721] | 0.747 | [0.719, 0.774] | 0.073 | [0.055, 0.090] | 0.356 | [0.126, 0.580] | 0.865 | [0.747, 0.994] |
| TUG | 0 | 0.796 | [0.749, 0.838] | 0.158 | [0.138, 0.179] | 0.707 | [0.670, 0.749] | 0.593 | [0.533, 0.650] | 0.791 | [0.718, 0.853] | 0.676 | [0.629, 0.722] | 0.083 | [0.055, 0.113] | 0.595 | [0.254, 0.973] | 1.034 | [0.818, 1.268] |
| TUG | 1 | 0.711 | [0.623, 0.792] | 0.183 | [0.147, 0.221] | 0.710 | [0.650, 0.771] | 0.527 | [0.425, 0.614] | 0.612 | [0.489, 0.723] | 0.745 | [0.675, 0.808] | 0.122 | [0.076, 0.170] | 0.144 | [-0.465, 0.704] | 0.633 | [0.350, 0.951] |
| TUG | 2 | 0.882 | [0.813, 0.941] | 0.074 | [0.052, 0.097] | 0.838 | [0.791, 0.883] | 0.551 | [0.416, 0.680] | 0.767 | [0.615, 0.909] | 0.848 | [0.799, 0.895] | 0.060 | [0.038, 0.086] | 0.268 | [-0.340, 0.929] | 1.282 | [0.906, 1.735] |
| TUG | 3 | 0.708 | [0.644, 0.766] | 0.168 | [0.144, 0.194] | 0.710 | [0.671, 0.751] | 0.455 | [0.378, 0.527] | 0.520 | [0.426, 0.617] | 0.768 | [0.723, 0.810] | 0.098 | [0.066, 0.133] | 0.094 | [-0.353, 0.555] | 0.658 | [0.435, 0.884] |
| WOMAC | Pooled | 0.704 | [0.679, 0.730] | 0.180 | [0.171, 0.189] | 0.663 | [0.644, 0.683] | 0.519 | [0.489, 0.549] | 0.634 | [0.598, 0.674] | 0.675 | [0.652, 0.698] | 0.042 | [0.032, 0.054] | 0.089 | [-0.061, 0.242] | 0.956 | [0.832, 1.088] |
| WOMAC | 0 | 0.698 | [0.651, 0.741] | 0.174 | [0.157, 0.191] | 0.652 | [0.612, 0.689] | 0.476 | [0.416, 0.536] | 0.597 | [0.526, 0.668] | 0.672 | [0.627, 0.714] | 0.049 | [0.033, 0.070] | -0.008 | [-0.276, 0.280] | 0.932 | [0.703, 1.160] |
| WOMAC | 1 | 0.651 | [0.596, 0.700] | 0.182 | [0.163, 0.200] | 0.691 | [0.654, 0.724] | 0.460 | [0.395, 0.529] | 0.498 | [0.421, 0.578] | 0.760 | [0.720, 0.800] | 0.080 | [0.052, 0.108] | -0.065 | [-0.423, 0.270] | 0.746 | [0.493, 0.980] |
| WOMAC | 2 | 0.748 | [0.690, 0.803] | 0.167 | [0.148, 0.187] | 0.653 | [0.603, 0.698] | 0.537 | [0.465, 0.601] | 0.749 | [0.667, 0.825] | 0.618 | [0.561, 0.674] | 0.072 | [0.045, 0.105] | 0.070 | [-0.277, 0.392] | 1.206 | [0.863, 1.593] |
| WOMAC | 3 | 0.731 | [0.681, 0.776] | 0.198 | [0.180, 0.217] | 0.651 | [0.605, 0.694] | 0.601 | [0.543, 0.657] | 0.736 | [0.671, 0.805] | 0.603 | [0.550, 0.654] | 0.067 | [0.042, 0.097] | 0.367 | [0.079, 0.656] | 1.137 | [0.858, 1.419] |
| EQ-5D-Index | Pooled | 0.770 | [0.749, 0.789] | 0.237 | [0.228, 0.245] | 0.655 | [0.636, 0.675] | 0.593 | [0.567, 0.619] | 0.469 | [0.441, 0.497] | 0.870 | [0.850, 0.892] | 0.192 | [0.172, 0.212] | 1.142 | [0.990, 1.309] | 1.289 | [1.157, 1.433] |
| EQ-5D-Index | 0 | 0.751 | [0.712, 0.786] | 0.237 | [0.220, 0.253] | 0.656 | [0.619, 0.691] | 0.634 | [0.588, 0.676] | 0.535 | [0.483, 0.588] | 0.809 | [0.760, 0.856] | 0.190 | [0.154, 0.231] | 0.958 | [0.721, 1.232] | 1.085 | [0.874, 1.295] |
| EQ-5D-Index | 1 | 0.751 | [0.711, 0.790] | 0.236 | [0.219, 0.253] | 0.645 | [0.605, 0.684] | 0.535 | [0.478, 0.590] | 0.407 | [0.351, 0.464] | 0.885 | [0.850, 0.920] | 0.169 | [0.133, 0.203] | 1.030 | [0.762, 1.309] | 1.302 | [1.035, 1.609] |
| EQ-5D-Index | 2 | 0.816 | [0.774, 0.854] | 0.211 | [0.190, 0.233] | 0.693 | [0.647, 0.736] | 0.537 | [0.465, 0.604] | 0.404 | [0.332, 0.471] | 0.922 | [0.886, 0.954] | 0.162 | [0.127, 0.201] | 1.373 | [0.944, 1.854] | 1.618 | [1.279, 2.013] |
| EQ-5D-Index | 3 | 0.757 | [0.715, 0.802] | 0.260 | [0.243, 0.277] | 0.633 | [0.591, 0.672] | 0.626 | [0.570, 0.675] | 0.489 | [0.431, 0.543] | 0.878 | [0.828, 0.921] | 0.262 | [0.226, 0.298] | 1.459 | [1.144, 1.812] | 1.314 | [1.035, 1.644] |

*Notes: AUC: Area under the receiver operating characteristics curve; CI: Confidence interval; ICI: Integrated Calibration Index; TUG: Timed Up and Go Test; WOMAC: Western Ontario McMaster University Osteoarthritis Index; EQ-5D-Index: EuroQol 5-Dimensions Questionnaire Index*

*Interpretation: AUC, Accuracy, F1: higher is better (maximum 1.0); Brier score, ICI: lower is better (minimum 0.0); Calibration intercept: ideal = 0; Calibration slope: ideal = 1.*

*^1^ Decision threshold set to outcome prevalences in the training data: TUG: 0.185; WOMAC: 0.265; EQ-5D-Index: 0.430*

## Table S4. Sensitivity analysis: Pooled model evaluation results.

| **Target** | **TUG (n=1466)** | | **WOMAC (n=2285)** | | **EQ-5D-Index (n=2285)** | |
| --- | --- | --- | --- | --- | --- | --- |
| **Algorithm** | **AG** | **LR** | **AG** | **LR** | **AG** | **LR** |
| **AUC**  **95%-CI** | 0.879  [0.861, 0.896] | 0.876  [0.858, 0.893] | 0.802  [0.783, 0.820] | 0.801  [0.782, 0.819] | 0.766  [0.746, 0.786] | 0.740  [0.720, 0.761] |
| **DeLong test**  **(*p*-value)^1^** | 0.358 | | 0.624 | | <0.001 | |
| **Brier score 95%-CI** | 0.139  [0.127, 0.152] | 0.141  [0.130, 0.151] | 0.179  [0.171, 0.188] | 0.190  [0.182, 0.197] | 0.186  [0.178, 0.195] | 0.188  [0.179, 0.197] |
| **Accuracy 95%-CI^2^** | 0.801  [0.780, 0.821] | 0.810  [0.789, 0.829] | 0.713  [0.693, 0.731] | 0.691  [0.671, 0.710] | 0.685  [0.666, 0.704] | 0.684  [0.665, 0.702] |
| **F1 95%-CI^2^** | 0.844  [0.826, 0.861] | 0.857  [0.840, 0.873] | 0.741  [0.721, 0.761] | 0.744  [0.725, 0.763] | 0.731  [0.713, 0.749] | 0.760  [0.742, 0.776] |
| **Sensitivity 95%-CI^2^** | 0.818  [0.794, 0.842] | 0.865  [0.843, 0.887] | 0.743  [0.718, 0.768] | 0.810  [0.789, 0.833] | 0.668  [0.644, 0.692] | 0.781  [0.758, 0.801] |
| **Specificity 95%-CI^2^** | 0.767  [0.732, 0.800] | 0.702  [0.665, 0.738] | 0.675  [0.647, 0.704] | 0.541  [0.509, 0.571] | 0.715  [0.682, 0.746] | 0.511  [0.476, 0.546] |
| **ICI 95%-CI** | 0.068  [0.052, 0.083] | 0.075  [0.061, 0.091] | 0.059  [0.047, 0.072] | 0.102  [0.089, 0.115] | 0.060  [0.046, 0.074] | 0.068  [0.054, 0.082] |
| **Calibration intercept 95%-CI** | 0.386  [0.234, 0.520] | 0.461  [0.305, 0.607] | 0.023  [-0.076, 0.119] | 0.152  [0.053, 0.252] | 0.297  [0.194, 0.398] | 0.163  [0.032, 0.280] |
| **Calibration slope 95%-CI** | 0.807  [0.737, 0.883] | 0.850  [0.727, 0.984] | 0.987  [0.905, 1.065] | 1.168  [1.047, 1.306] | 0.886  [0.803, 0.976] | 0.745  [0.656, 0.844] |

*Notes: LR: Baseline logistic regression; AG: Multivariable ensemble AutoGluon model; AUC: Area under the receiver operating characteristics curve; CI: Confidence interval; ICI: Integrated Calibration Index; TUG: Timed Up and Go Test; WOMAC: Western Ontario McMaster University Osteoarthritis Index; EQ-5D-Index: EuroQol 5-Dimensions Questionnaire Index*

*Interpretation: AUC, Accuracy, F1: higher is better (maximum 1.0); Brier score, ICI: lower is better (minimum 0.0); Calibration intercept: ideal = 0; Calibration slope: ideal = 1.*

*^1^ A p-value < 0.05 indicates a significant difference between the AUCs of the multivariable ensemble and baseline logistic regression model*

*^2^ Decision threshold set to outcome prevalences in the training data: TUG: 0.525; WOMAC: 0.445; EQ-5D-Index: 0.611*

## Figure S1: Receiver operating characteristics curves.

*Notes: TUG: Timed Up and Go Test; WOMAC: Western Ontario McMaster University Osteoarthritis* *Index; EQ-5D-Index: EuroQol 5-Dimensions Questionnaire Index*

## Figure S2: Confusion matrices (normalized).

Notes: TUG: Timed Up and Go Test; WOMAC: Western Ontario McMaster University Osteoarthritis Index; EQ-5D-Index: EuroQol 5-Dimensions Questionnaire Index

Decision threshold set to respective outcome prevalences in the training data: TUG: 0.185; WOMAC: 0.265; EQ-5D-Index: 0.430

## Figure S3: Sensitivity and specificity across decision thresholds for predicting non-response.

*Notes: TUG: Timed Up and Go Test; WOMAC: Western Ontario McMaster University Osteoarthritis Index; EQ-5D-Index: EuroQol 5-Dimensions Questionnaire Index*

*Decision threshold set to outcome prevalences in the training data: TUG: 0.185; WOMAC: 0.265; EQ-5D-Index: 0.430*

## Figure S4: Calibration plots.

*Notes: TUG: Timed Up and Go Test; WOMAC: Western Ontario McMaster University Osteoarthritis Index; EQ-5D-Index: EuroQol 5-Dimensions Questionnaire Index*

## Figure S5: Decision curve analyses.

*Notes: TUG: Timed Up and Go Test; WOMAC: Western Ontario McMaster University Osteoarthritis Index; EQ-5D-Index: EuroQol 5-Dimensions Questionnaire Index*

## Supplementary Methods

### Software specifications

| **Software/Library** | **Version** |
| --- | --- |
| autogluon | 1.4.0 |
| Ipython | 8.27.0 |
| jupyter lab | 4.2.5 |
| matplotlib | 3.9.2 |
| mlstatkit | 0.1.9 |
| numpy | 2.1.1 |
| pandas | 2.3.3 |
| python | 3.12.3 |
| scikit-learn | 1.6.1 |
| shap | 0.48.0 |

### Characteristics of inpatient rehabilitation treatment in Austria

All patients participated in a structured, interdisciplinary three-week inpatient rehabilitation programme in accordance with current evidence-based guidelines and the Austrian medical service profile for rehabilitation [1,2]. The programme comprised at least 1,800 minutes of active and passive therapy over three weeks, corresponding to approximately 2–3 hours of therapy per day.

Active therapies included individual physiotherapy, medical exercise therapy, aquatic exercise, endurance training (e.g. cycling and Nordic walking), and strength and balance exercises. Passive or adjunctive treatments included lymphatic drainage, medical massage, electrotherapy, ultrasound therapy, and stretching or relaxation techniques. Additionally, patients received health education and lifestyle counselling aimed at promoting long-term self-management.

In Austria, follow-up rehabilitation (post-acute therapy in specialized rehabilitation centers) is generally required to commence within three months after the acute event or surgery. For the training dataset, where these data were available, the median time between surgery and the start of rehabilitation was 72 days, which is consistent with earlier findings where a mean of 66.4 was reported [3].

1.	Grote V, Unger A, Puff H, Böttcher E. What to Expect: Medical Quality Outcomes and Achievements of a Multidisciplinary Inpatient Musculoskeletal System Rehabilitation. In: Bernardo-Filho M, Cunha De Sá-Caputo D, Taiar R, editors. Physical Therapy Effectiveness [Internet]. IntechOpen; 2020 [cited 2026 Jul 7]. Available from: https://www.intechopen.com/books/physical-therapy-effectiveness/what-to-expect-medical-quality-outcomes-and-achievements-of-a-multidisciplinary-inpatient-musculoske doi:10.5772/intechopen.89596

2.	Pensionsversicherungsanstalt. Medizinisches Leistungsprofil der Pensionsversicherung – Neue Maßstäbe in der Rehabilitation [Internet]. [cited 2026 Jul 7]. Available from: <https://www.pv.at/web/reha-und-praevention/medizinisches-leistungsprofil>

3.	V. Grote et al., “General and Disease-Specific Health Indicator Changes Associated with Inpatient Rehabilitation,” J. Am. Med. Dir. Assoc., vol. 21, no. 12, p. 2017.e10-2017.e27, Dec. 2020, doi: 10.1016/j.jamda.2020.05.034.
